# Metallic foreign body in the ulnar nerve in the Guyon’s canal and Review of the Literature

**DOI:** 10.1080/23320885.2021.1939702

**Published:** 2021-06-18

**Authors:** Marco Guidi, Stefano Lucchina, Bong-Sung Kim, Inga Besmens, Paolo Ivan Fiore, Nicola Altin, Alan Cortesi, Nora Huber, Maurizio Calcagni, Martin Riegger

**Affiliations:** a Department of Plastic Surgery and Hand Surgery, University Hospital Zurich, Zurich, Switzerland; b Hand Surgery Unit, Locarno’s Regional Hospital, Locarno, Switzerland; c Locarno Hand Center, Locarno, Switzerland; d Orthopaedic and Traumatology Department, Regional Hospital San Giovanni, Bellinzona, Switzerland

**Keywords:** Foreign body, ulnar nerve, nerve injury

## Abstract

The authors present a rare case of a 61-year-old patient with a metal foreign body inside the ulnar nerve in the Guyon's canal. After the surgical removal the motor function was not impaired. At 6-month follow up from the surgical removal, the patient showed a complete motor and sensory function.

## Introduction

Accidental penetration of foreign bodies is a common occurrence and usually not associated with complications. A potential nerve lesion represents a clinical indication for surgical revision, visualization and microsurgical repair.

To our best knowledge there are few reports in the literature of missed foreign bodies in the medial nerve in the wrist region with associated nerve compression or injury [1–4]. So far, there are no reports of penetrating foreign body of the ulnar nerve at the wrist.

The authors present a rare case of a patient with a metal body penetrating into the ulnar nerve inside the Guyon's canal after an accidental fall.

## Case report

A 61-year-old female patient, chronic alcoholic and heavy smoker, was referred to the Emergency department after an accidental fall the day before, due to alcohol abuse. The patient reported pain in her left hand where she sustained an injury proximal to the volar crease of the wrist.

The hand had an 8 mm wound with perilesional erythema. The hand was painful on the hypothenar eminence with hypoesthesia on the ulnar side of the ring finger and on the radial side of the little finger. Static 2-point discrimination (S-2PD) was assessed with a Mackinnon-Dellon Disk-Criminator™ (Sensory Management, Towson, USA) and was equivalent to 12 mm in the area of the common palmar digital branch of the ulnar nerve. No motory deficits were present.

The radiography of the hand showed no fractures with the presence of a metal foreign body at the level of the hypothenar eminence. Blood tests showed a leukocytosis of 12730 G/L and a C-reactive protein (CRP) of 100 mg/L.

Because of the suspicion of wound infection, surgery was performed two hours after admission to the emergency room with the use of microsurgical loupes under 3.5× magnification and locoregional anesthesia of the brachial plexus.

**Figure 1.**
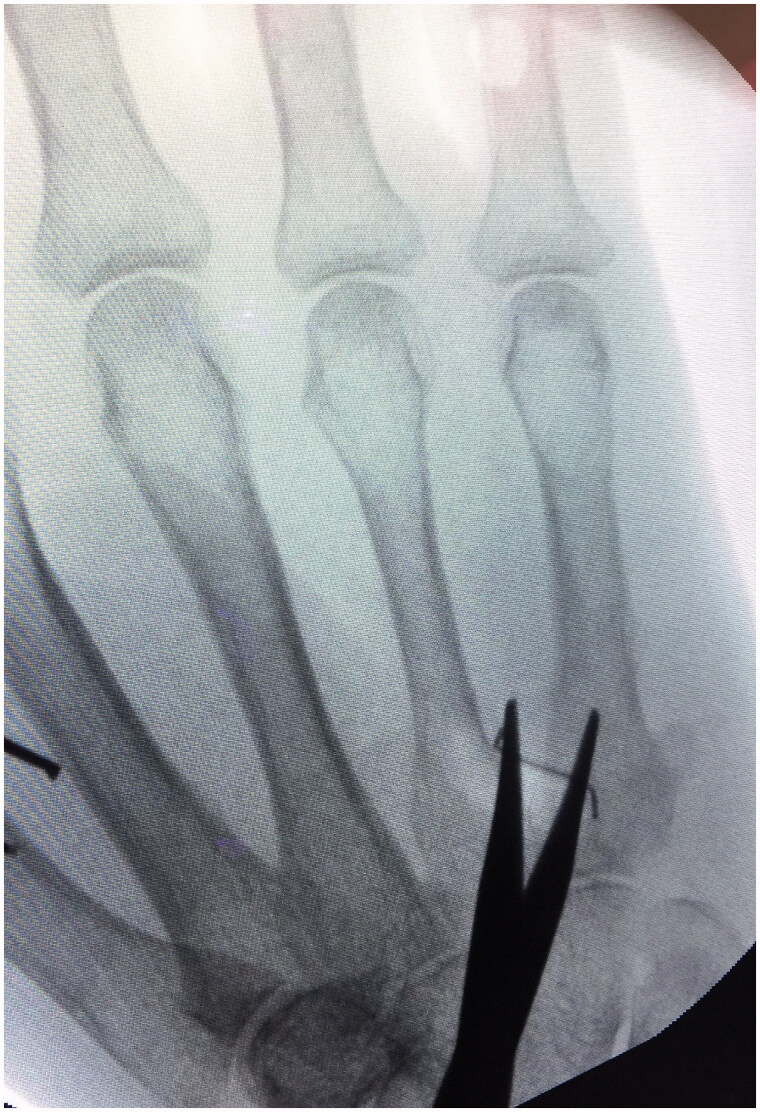
Intraoperative detection of the foreign body under fluoroscopy with the help of a dissecting scissor.

**Figure 2.**
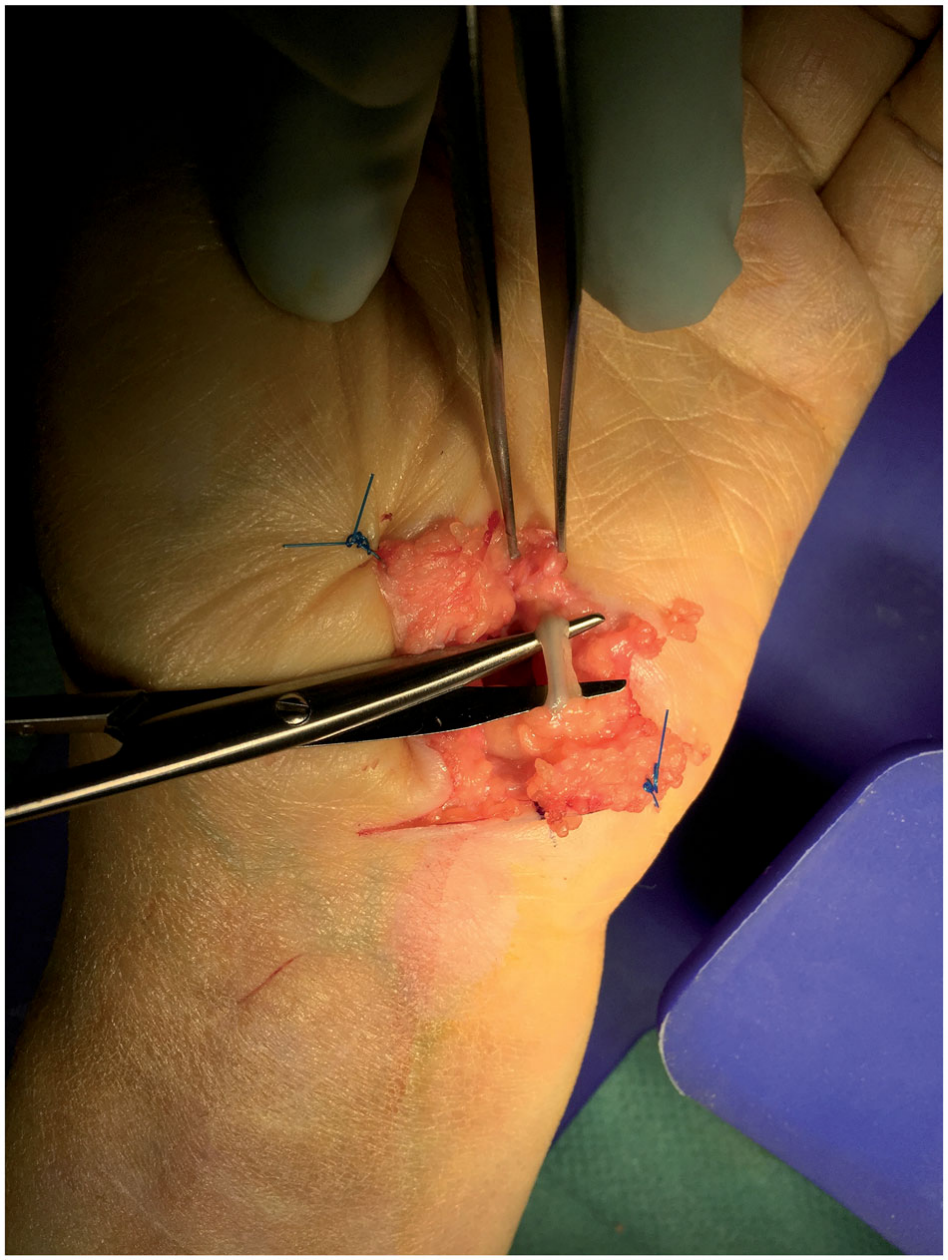
Appearance of the foreign body inside the ulnar nerve above the dissecting scissors in the Guyon’s canal.

**Figure 3.**
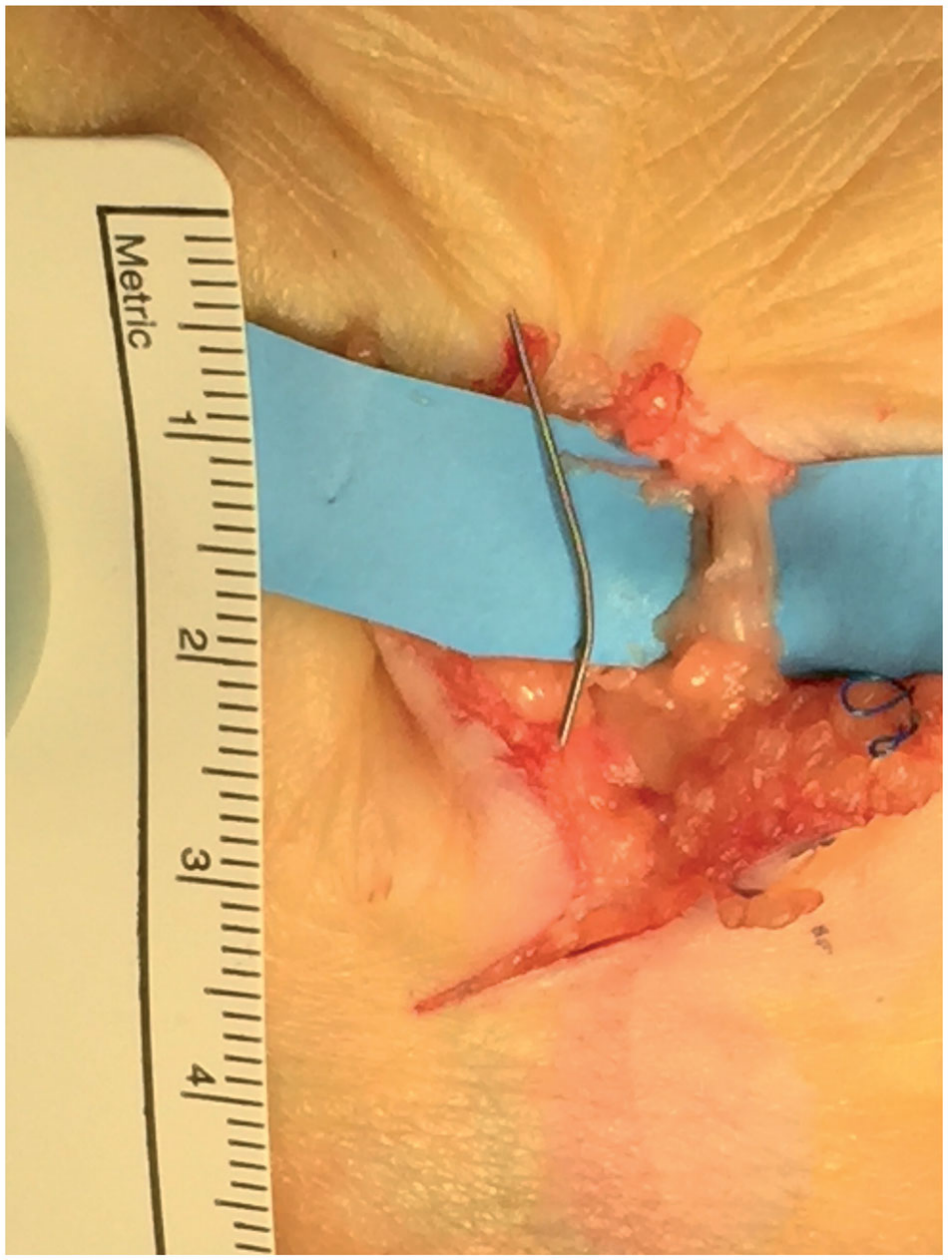
Metallic foreign body removal (20 m × 1 mm).

**Figure 4.**
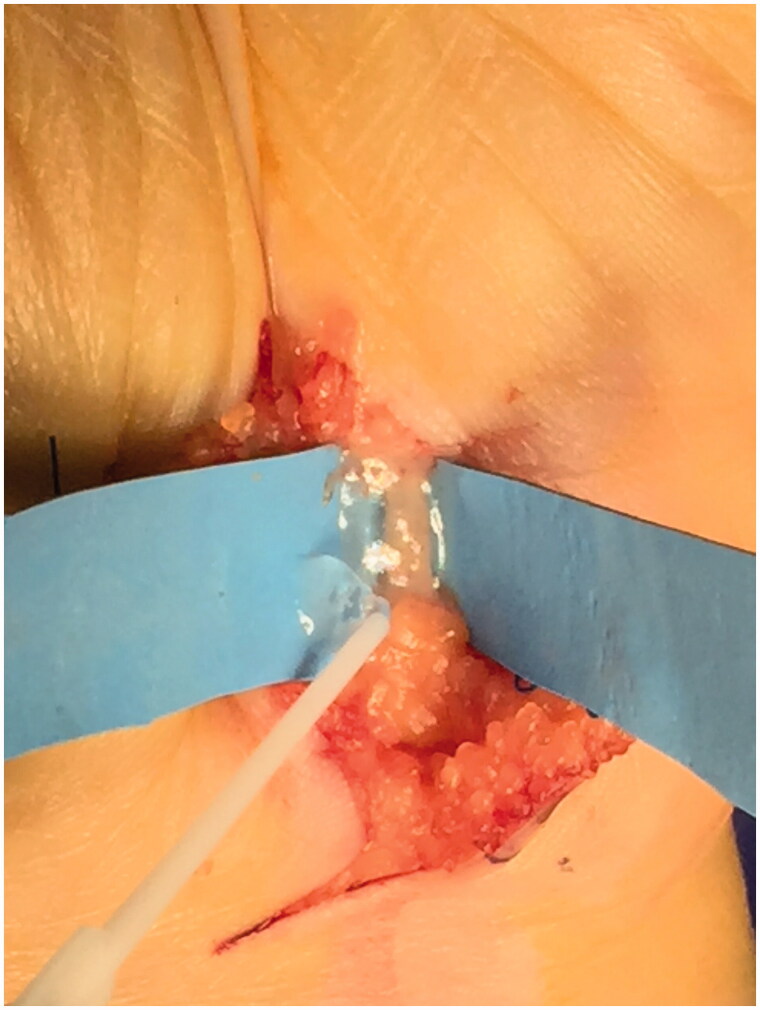
Ulnar nerve closure with fibrin glue.

During the exploration of the wound, it was not possible to detect the foreign body in the subcutaneous fat. After opening the Guyon's canal, the foreign body was located easily under fluoroscopy medial to the ulnar artery (Figures 1 and 2). The ulnar nerve in the Guyon’s canal had been injured by the foreign body that was detectable between the fascicles. The ulnar artery was intact. A small section of the epineurium (Figure 3) of the ulnar nerve was made and the foreign body removed. The nerve then was reconstructed under microscope with fibrin glue (Figure 4).

A “custom made” palmar thermoplastic splint was worn for pain control for 48 h then a free active range of motion of wrist and long fingers was reinforced.

No complications were detectable in the post-operative phase. At 6-months follow up post-operatively the hand had an extrinsic and intrinsic muscles’ function with a complete closure of the fist. The S-2PD was 5 mm in the 4th and 5th finger. The handgrip strength with a Jamar dynamometer reached 80% of the force of the unaffected side.

## Discussion

Due to the continuous use of the hands in daily and work activities, the chance of sustaining injuries with the penetration of foreign bodies is very high. The suspicion of foreign bodies should always be assessed in case of injuries involving glass, metal fragments, wooden materials, accidental falls. Radiography may be used to locate foreign bodies for removal and ultrasonography can be a helpful tool for localizing radiolucent foreign bodies [5]. Although these injuries may seem minor, wound with neglected foreign bodies are a common cause of malpractice claims [6]. Inert metal foreign bodies may not have to be removed, because removal might cause more trauma than simply leaving them in place. Therefore, the decision to remove a foreign body is based on symptoms or risk of complications [5]. For wounds that require imaging, appropriate modalities include radiography, computed tomography (CT), and ultrasonography, depending on the size and type of foreign body. Plain radiography is the most economic and available method for viewing radiopaque foreign bodies, including metal [7]. Although CT is more sensitive than radiography, the increased cost limits CT to foreign bodies that are not visible on radiography [8].

Ultrasonography is fast and low-cost and helpful in finding radiolucent foreign bodies [9]. Detection of foreign bodies with ultrasonography has a sensitivity of 50 to 90 precent and a specificity of 70 to 97 percent for metal and plastic fragments [7]. Ultrasonography can help determine the depth, size and shape of the foreign body and its relationship to anatomic structures such as bones, tendons, vessels, nerves or joints [9]. Many foreign bodies are surrounded by a hypoechogenic area representing inflammation and helping the physician to localize the foreign bodies with smaller skin incisions [9].

Anderson et al. [10] described 200 consecutive patients with foreign bodies in the hand and in 38% the diagnosis was missed by the physician initially. Salati et al. [11] in their report of 61 cases with foreign bodies penetrating the hand, showed that 21% were metal fragments and there was a low risk of nerve injury (1% median nerve). In the literature there are isolated cases reports of nerve compression or injury following penetration of foreign bodies in the wrist [1–4]. Lesions of the median nerve have already been described in some papers following the migration of Kirschner wires or foreign bodies [3]. Southworth [4] described the presence of a fragment of a needle in the median nerve after an acupuncture session.

Kitay and Wolfe [12] first described a lesion of the deep motor branch of the ulnar nerve in the wrist by a retained foreign body. Pleser et al. [13] they first reported the presence of a foreign body within the ulnar nerve in the arm that was surgically removed. To our best knowledge, this is the only description after Pleser and collegues [13] of a percutaneously penetrated metallic foreign body with an intraneural location. There are no other cases in the literature of foreign bodies inside the ulnar nerve at the wrist. Therefore, the case described in this article represents the first documented case of the presence of a metal fragment inside the ulnar nerve in the Guyon’s canal.

This rare injury must be treated surgically to perform a nerve repair and foreign body removal and to prevent the risk of infection [9] that is usually higher in lacerations in older patients and those with diabetes and in wound that are longer, wider, deeper, jagged and with associated visible contamination [14].

## Conclusions

Isolated penetration of peripheral nerve due to retained foreign bodies are very rare, but increased awareness of this eventuality will help avoid future delays in diagnosis and treatment. Prompt treatment with foreign body removal and microsurgical repair of the injured nerve can ensure a fast functional and clinical recovery.

## Statement of informed consent

Informed consent was obtained from the patient of the study according to the Declaration of Helsinki.

## Statement of human and animal rights

No experiments on animals were performed for this study. No experimental procedures were performed in any human subject for this study.
